# The complete chloroplast genome of the *Syzygium buxifolium* Hook. Et Arn.1833 and its phylogenetic analysis

**DOI:** 10.1080/23802359.2024.2371553

**Published:** 2024-06-27

**Authors:** Fang Liu, Lichai Yuan, Yuanfang Zhang

**Affiliations:** a Xiangnan University, Chenzhou, P.R. China; b Institute of Medicine Plant Development, Chinese Academy of Medical Sciences and Peking Union Medical College, Beijing, P.R. China

**Keywords:** Next-generation sequencing, plastome, genome assembly and annation, phylogeny

## Abstract

*Syzygium buxifolium.* Hook. Et Arn.1833 is a member of the Myrtaceae family. This species is used in traditional Chinese medicines. It possesses numerous synonyms, reflecting the ambiguity in its taxonomy. The chloroplast genome has been widely used for species identification and phylogenetic analysis. Regrettably, there is a lack of information regarding the chloroplast genome of *S. buxifolium*. Here, we intend to obtain the chloroplast genome of *S. buxifolium* to resolve its classification problems. In particular, we utilized Illumina sequencing technology to sequence, GetOrganelle to assemble, and CPGAVAS2 to characterize the chloroplast genome of *S. buxifolium*. The chloroplast genome of *S. buxifolium* had a length of 158,581 bp and consisted of 111 unique genes, comprising 78 protein-coding genes, 29 transfer RNA (tRNA) genes, and four ribosomal RNA (rRNA) genes. In addition, we identified 86 Simple Sequence Repeats, 345 tandem repetitive sequences, and 34 dispersed repetitive sequences using modules implemented in CPGAVAS2. Lastly, we carried out phylogenetic analysis using Phylosuite. The results indicated a close relationship between *S. buxifolium* and *S. grijsii*. This study offers novel genetic data for the molecular identification and subsequent phylogenetic analysis of the *Syzygium* genus.

## 1. Introduction

*Syzygium buxifolium* Hook. Et Arn.1833 (Schoch et al. 2020), a member of the Myrtaceae family, is a plant species found in sparse woods or shrub communities in low mountainous regions. It may grow up to 5 meters tall and is widely distributed in Guangdong, Hunan, Fuzhou, Guizhou, and other locations (Song et al. 1999). The complete plant of *S. buxifolium* has been utilized medicinally. Its roots can treat asthma, encourage dampness, strengthen the spleen, and disperse blood stasis, while its leaves cleanse and purify (Zhao et al. 2006). In traditional Chinese medicines, *S. buxifolium* is mainly used to treat asthma, cancer spread, swelling, discomfort, burns, falls, swelling, and other diseases (Qin et al. 2002; Zhao et al. 2006). *S. buxifolium’*s primary bioactive elements are flavonoids, triterpenes, sterols, and essential oils (Zhou et al. 1998; Huong et al. 2023). Pharmacological studies have revealed that *S. buxifolium* has antibacterial, antiviral, antioxidant, anticancer, anti-aging, hypotensive, lipid-lowering, and immunomodulatory properties (Chen et al. 2016; Ding et al. 2018). However, due to their similar morphologies, *Syzygium grijsii* (Hance) Merr. & L.M. Perry and *S. buxifolium* are often confused (Huang 2003). Accurate identification of *S. buxifolium* materials is essential to ensure the efficacy and safety of their medicinal products.

The chloroplast is a plant organelle containing multiple essential proteins involved in photosynthesis and other metabolic processes (Keeling 2004). The chloroplast genome typically consists of four components: a large single-copy (LSC), a short single-copy (SSC), and two inverted repeats (IRs) regions (Palmer 1991; Wicke et al. 2011). The chloroplast genome is smaller than the nuclear and mitochondrial genomes and is not prone to recombination and nucleotide changes (Daniell et al. 2016). Phylogenetic analyses utilizing protein-coding and whole chloroplast genomes provide novel perspectives and significant advancements in our comprehension of plant evolution (Asaf et al. 2020). The non-coding region of chloroplast genomes has been used for plant species identification (Ngai et al. 2023). Nevertheless, the chloroplast genome of *S. buxifolium* has yet to be sequenced.

This study aims to sequence, assemble, and annotate the chloroplast genome of *S. buxifolium* in order to resolve taxonomic confusion. Additionally, by utilizing the chloroplast genome data to construct a phylogenetic tree, this research provides reliable and accurate genetic data support for species identification, molecular marker development, and systematic evolutionary studies of the genus *Syzygium*.

## 2. Materials and methods

**Figure 1.**
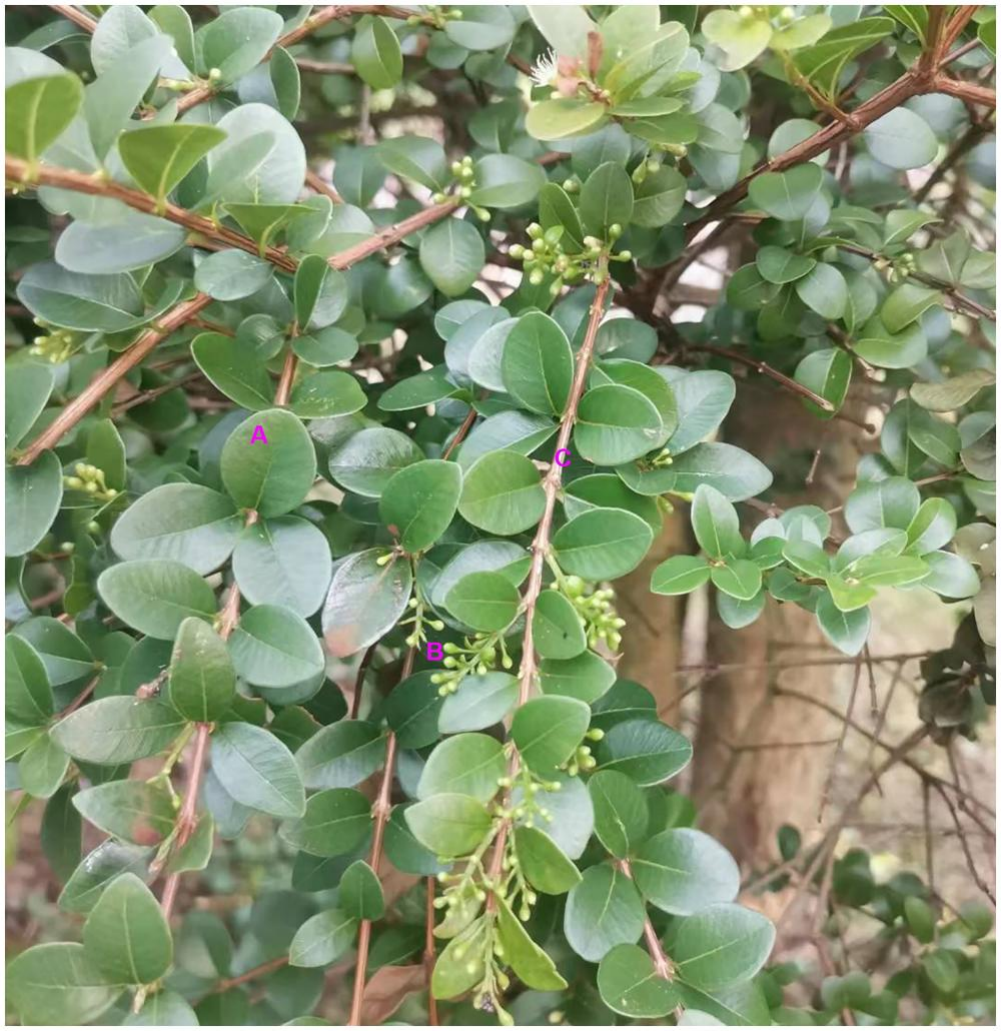
Photo of *S. buxifolium*. Lichai Yuan took the picture at Zhaiqian town, Guidong County, Chenzhou City, Hunan Province, China (N25˚59′26ʺ, E113˚54′39ʺ). A: Leaf; B: Inflorescence; C: branchlet.

Fresh leaves of *S. buxifolium* were gathered in Zhaiqian Town, Guidong County, Chenzhou City, Hunan Province, China (N25˚59′26ʺ, E113˚54′39ʺ) (Figure 1). Healthy, pest-free mature leaves were picked, rinsed with tap water, dried, and utilized to extract DNA using the CTAB technique. The leftover leaves were covered in aluminum foil and stored at −80 °C. One sample was deposited at the Institute of Medicinal Plant Development (http://www.implad.ac.cn/, lcyuan2008@163.com) with the voucher number 20230711027.

We fragmented the genomic DNA into roughly 500 bp long insert fragments. We then used the NEBNext Ultra library construction kit for Illumina to construct the sequencing library. The library was sequenced on an Illumina platform using the PE150 mode at Novogene Bioinformatics Technology Co. Ltd. (Beijing, China). We used Trimmomatic (v0.35) (v0.35) (Bolger et al. 2014) to filter the raw data and eliminate adapters and low-quality bases. Clean data were assembled using the GetOrganelle v1.7.5 software (Jin et al. 2020). Gepard (Krumsiek et al. 2007) aligned the chloroplast genome to *Arabidopsis thaliana* (NC_000932.1). We used CPGAVAS2 (Shi et al. 2019) to annotate the chloroplast genome and manually corrected the annotation results using Apollo (Lee et al. 2009). The circular plastome was visualized using the CPGview tool (Liu et al. 2023).

Then, using the PhyloSuite program (Zhang et al. 2020) (Table S1), we built two trees using the whole chloroplast genome sequences of *S. buxifolium* and 17 other *Syzygium* species, with *Eugenia reinwardtiana* (Blume) DC (NC_084367.1) as outgroup. We used MAFFT to align these nucleic acid sequences (Katoh et al. 2019). Next, we built a tree using the maximum likelihood (ML) method implemented in IQ-tree v. 1.6.12 (Nguyen et al. 2015). In parallel, we constructed the Bayesian inference (BI) tree. The ML tree was built with the TVM + F + I + G4 model, and the BI tree was constructed with the GTR + I + G model. Both models were selected based on BIC. Lastly, the phylogenetic trees were shown using iTol (Letunic and Bork 2021).

## 3. Results

A total of 10.78 GB of raw data was produced. After filtering, 10.71 GB of clean data was acquired. We assembled the sequencing data and obtained two circular sequences of the *S. buxifolium’*s chloroplast genome. The two sequences differed in the SSC region’s orientation. The two sequences were compared with the chloroplast genome of *Arabidopsis thaliana*. For downstream analysis, we selected the sequence whose SSC region’s orientation is identical to that of the *A. thaliana* (Figure S1).

To validate the correctness of the assembled genome sequence, we mapped the sequencing reads to the assembled sequence. We obtained a 1527 × to 5654 × depth across the assembled genome, with an average depth of 3703.39 × (Figure S2). The results demonstrated the dependability of the assembly result.

**Figure 2.**
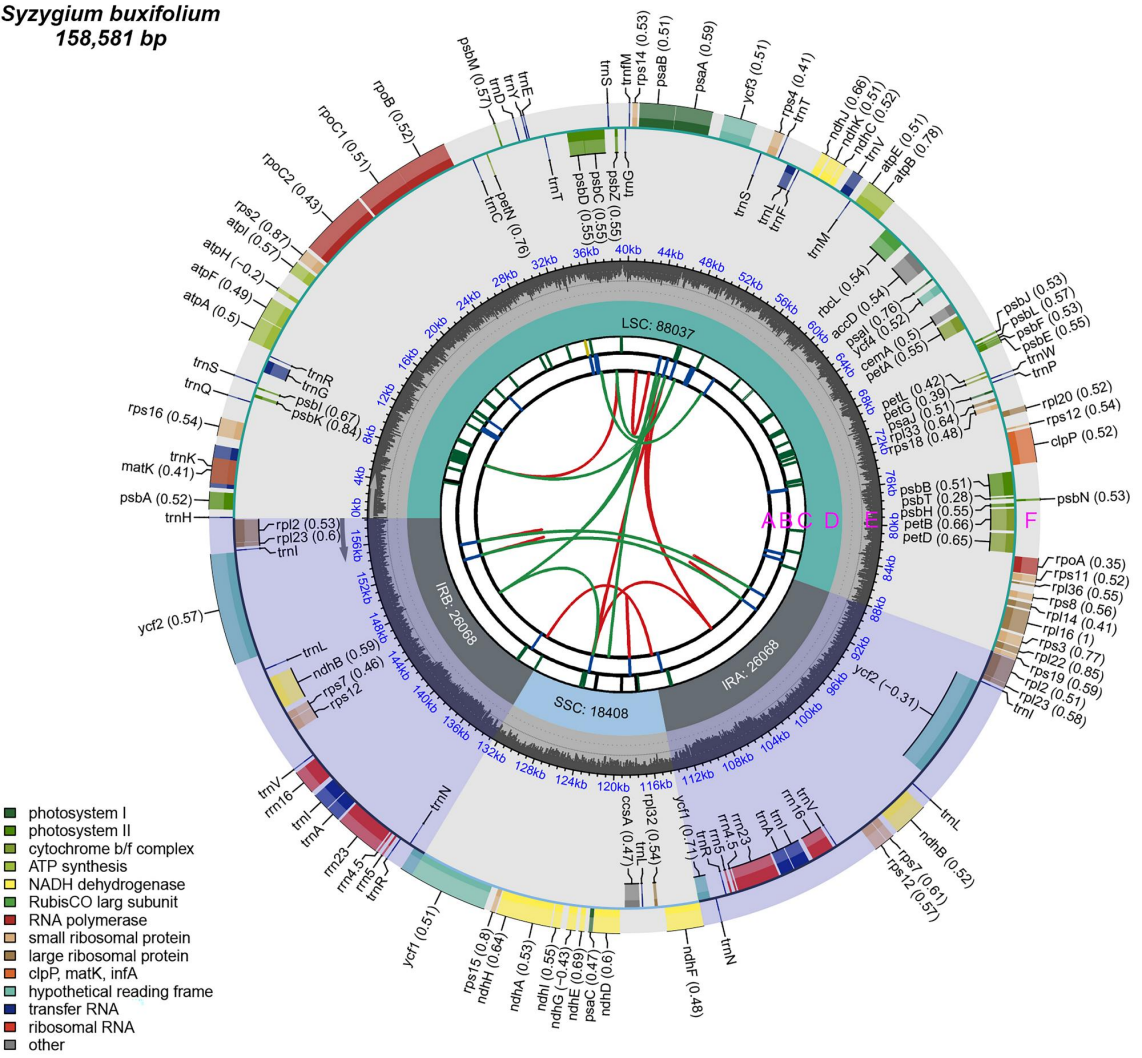
The genomic map of the chloroplasts in *S. buxifolium*. There are six tracks on the map. The first track (A) displays the forward and backward repeats connected by red and green arcs, viewed from the center outward. The tandem repeats are displayed as brief blue bars on track B, the second track. The microsatellite sequences are displayed as brief green and yellow bars on the third track (C). On the fifth track (D), the small single-copy (SSC), inverted repeat (IRa and IRb), and large single-copy (LSC) areas are displayed. Plotting of the GC content across the genome is done on track six (E). Plotting of genes is done on track seven (F). The gene name is followed by parenthesis with the optional codon use bias. Depending on how they are classified functionally, genes are colored. Transcription proceeds clockwise and counterclockwise for the inner and outer genes, respectively. The lower left corner displays the genes’ functional categorization.

With a total length of 158,581 bp, the chloroplast genome of *S. buxifolium* was a circular sequence with a conserved quadripartite structure (Figure 2). It was composed of two inverted repeat regions (26,069 bp), a large single copy region (88,036 bp), and a small single copy region (18,407 bp). The chloroplast genome had a total G/C composition of 36.76%. The LSC region’s G/C composition (34.85%) was notably higher than that of the SSC region (30.94%) but lower than that of the IR regions (42.75%) (Table S2).

The chloroplast genome of *S. buxifolium* encoded 111 unique genes, including four rRNA genes, 29 tRNA genes, and 78 unique protein-coding genes. Two protein-coding genes (*ycf*3 and *clp*P) included two introns, whereas twelve protein-coding genes (*atp*F, *ndh*A, *ndh*B (x2), *pet*B, *pet*D, *rpl*16, *rpl*2 (×2), *rpo*C1, *rps*16, and *ycf*1) contained one intron each. (Figure S3). Trans-splicing was applied to the *rps*12 genes (Figure S4). Furthermore, one intron was present in eight tRNA-coding genes (*trn*A-UGC (×2), *trn*G-UCC, *trn*I-GAU (×2), *trn*K-UUU, *trn*L-UAA, and *trn*V-UAC) (Table S3). The *S. buxifolium* chloroplast genome’s coding sequence (CDS), tRNA, and rRNA lengths were 79,413, 9,052, and 2,705 bp, respectively. These lengths accounted for 50.08%, 5.71%, and 1.71% of the genome’s overall length (Table S2).

**Figure 3.**
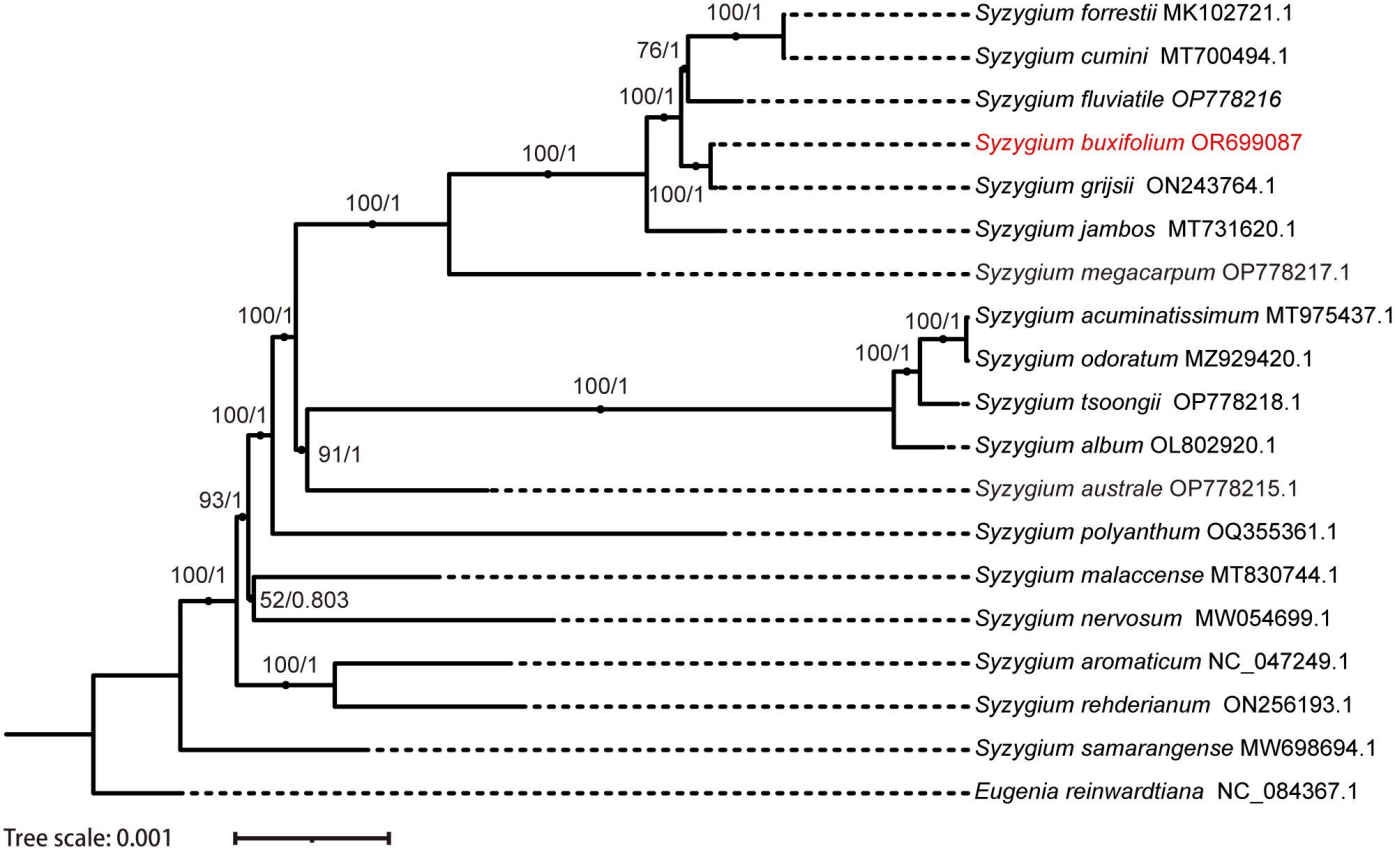
Phylogenetic tree generated using maximum likelihood (ML) Bayesian interference (BI) and based on the chloroplast genome. ML bootstrap (BS) and BI posterior probability (PP) values are represented by numbers at the nodes. The 17 *Syzyglium* were downloaded from GenBank, namely *S. forrestii (*MK102721.1) (Zhang et al. 2018), *S. cumini* (MT700494.1), *S. fluviatile* (OP778216.1), *S. buxifolium* (OR699087), *S. grijsii* (ON243764.1)*, S. jambos* (L.) Alston (MT731620.1) (Sun et al. 2020)*, S. megacarpum* Rathakr. & N. C. Nair (OP778217.1)*, S. aromaticum* (L.) Merr. & L.M.Perry (NC_047249.1)*, S. rehderianum* Merr. & L. M. Perry (ON256193.1), *S. samarangense* (Blume) Merr. & L. M. Perry (MW698694.1) (Wei et al. 2022), *S. nervosum* DC. (MW054699.1) (Li et al. 2021)*, S. malaccense* (L.) Merr. & L. M. Perry (MT830744.1), *S. polyanthum* (Wight) Walp. (OQ355361.1), *S. australe* (J. C. Wendl. ex Link) B. Hyland (OP778215.1), *S. acuminatissimum* (Blume) DC. (MT975437.1) (Zeng et al. 2021), *S. odoratum* (Lour.) DC. (MZ929420.1) (Chen et al. 2022), *S. tsoongii* (Merr.) Merr. & L. M. Perry (OP778218.1), *S. album* Q. F. Zheng (OL802920.1), *Eugenia reinwardtiana* (NC_084367.1).

To learn more about the phylogenetic relationship among the members of the *Syzygium* genus. We built phylogenetic trees using Maximum Likelihood (ML) and Bayesian Inference (BI) methods with the chloroplast genome sequences. The ML and BI approaches produced two trees with nearly identical topology. Both trees were supported with high bootstrap values. According to the phylogenetic tree, *S. buxifolium* and *S. grijsii* (Hance) Merr. & L. M. Perry formed a minor branch apart from *S. cumini* (L.) Skeels, *S. forrestii* Merr. & L. M. Perry, and *S. fluviatile* (Hemsl.) Merr. & L. M. Perry. The phylogenetic tree’s strong bootstrap scores for each branch node within the genus *Syzygium* (Figure 3).

## 4. Discussion and conclusions

In this study, we obtained the first chloroplast genome of *S. buxifolium* and characterized its genomic features. We determined the phylogenetic relationship between *S. buxifolium* and other species of *Syzygium.* We found it was closest to *S. grijsii*. Our study offers vital data for species identification and evolutionary analysis within the *Syzygium* genus.

The chloroplast genome possesses a conservative quadripartite structure spanning a length of 158,581 bp and harboring 111 distinct genes. The genome size of *S. buxifolium* is within the range of chloroplast genomes in other *Syzygium* species, which varies from 159,352 to 160,327 bp (Table S1) (Zhang et al. 2018; Sun et al. 2020; Chen et al. 2022; Wei et al. 2022).

The phylogeny study demonstrated a close genetic relationship between *S. buxifolium* and *S. grijsii*. This finding was consistent with the conventional morphological classification (Chase et al. 2016). Although the chloroplast genomes of both *S. buxifolium* (158,581 bp) and S. grijsii 158,591 bp) encoded the same numbers and types of genes and differed by only ten bases across the genome. In addition, we observed variation in several regions (e.g. *trn*G*-trn*F, *trn*E*-trn*T, *ycf3*, and *rps*32-*trn*F) (data not shown). Variations in these highly variable regions could be used for species identification. Unfortunately, we could not collect samples of *S. grijsii* to validate these highly variable regions experimentally, and we will next collect samples of *S. grijsii* for PCR validation.

In conclusion, we sequenced and characterized the chloroplast genome of *S. buxifolium.* Through phylogenetic analysis, we resolved the phylogenetic uncertainty of *S. buxifolium.* Lastly, we identified several highly variable regions that can be used for molecular marker development.

## Data Availability

The complete chloroplast genome sequences of *S. buxifolium* in this study have been submitted to the NCBI database under the accession number OR699087. The associated BioProject, Bio-Sample, and SRA numbers are PRJNA1055315, SAMN38989340, and SRS19952658.
